# The Mixed Nature of Incentives for Community Health Workers: Lessons from a Qualitative Study in Two Districts in India

**DOI:** 10.3389/fpubh.2016.00038

**Published:** 2016-03-14

**Authors:** Enisha Sarin, Sarah Smith Lunsford, Ankur Sooden, Sanjay Rai, Nigel Livesley

**Affiliations:** ^1^University Research Co., LLC, New Delhi, India; ^2^EnCompass LLC, Bethesda, MD, USA

**Keywords:** community health workers, incentives and rewards, India, performance, motivation

## Abstract

Incentives play an important role in motivating community health workers (CHWs). In India, accredited social health activists (ASHAs) are female CHWs who provide a range of services, including those specific to reproductive, maternal, neonatal, child, and adolescent health. Qualitative interviews were conducted with 49 ASHAs and one of their family members (husband, mother-in-law, sister-in-law, or son) from Gurdaspur and Mewat districts to explore the role of family, community, and health system in supporting ASHAs in their work. Thematic analysis revealed that incentives were both empowering and a source of distress for ASHAs and their families. Earning income and contributing to the household’s financial wellbeing inspired a sense of financial independence and self-confidence for ASHAs, especially with respect to relations with their husbands and parents-in-law. In spite of the empowering effects of the incentives, they were a cause of distress. Low incentive rates relative to the level of effort required to complete ASHA responsibilities, compounded by irregular and incomplete payment, put pressure on families. ASHAs dedicated much of their time and own resources to perform their duties, drawing them away from their household responsibilities. Communication around incentives from supervisors may have led ASHAs to prioritize and promote those services that yielded higher incentives, as opposed to focusing on the most appropriate services for the client. ASHAs and their families maintained hope that their positions would eventually bring in a regular salary, which contributed to retention of ASHAs. Incentives, therefore, are both motivating and inspiring as well as a cause dissatisfaction among ASHAs and their families. Recommendations include revising the incentive scheme to be responsive to the time and effort required to complete tasks and the out-of-pocket costs incurred while working as an ASHA; improve communication to ASHAs on incentives and responsibilities; and ensure timely and complete payment of incentives to ASHAs. The findings from this study contribute to the existing literature on incentivized CHW programs and help throw added light on the role incentives play in family dynamics which affects performance of CHW.

## Introduction

Community health worker (CHW) programs have been introduced as a strategy to link the community with the health care system with the aim to identify health needs of the community and bring these to the focus of health care providers. CHWs are lay health workers trained to deliver certain health services but have no formal professional education (1). They are paid for performing certain activities either as a salary or an honorarium (2) by a government health structure or non-governmental organizations (NGOs).

A review of incentives for CHWs found that they improve retention of workers (3). However, there are challenges as payments may be insufficient considering the level of effort required to complete the work, or may not be paid regularly (3). In general, CHWs earn less than a living wage and may resort to other sources of generating income (4, 5). In addition, incentives can influence CHWs to focus on funded activities at the expense of unfunded activities (6) and can lead to mistrust in the communities as clients suspect CHWs push particular services rather than promoting what is best for the client (7). Incentives can also lead communities to view CHWs as government employees and either expect more services than what CHWs can provide or lose trust in them (3). It has also been demonstrated that non-financial factors, such as training and supervision, job aids, improved working conditions, and personal growth opportunities, are important in CHW programs and should be considered when designing incentive programs (3, 7, 8).

## India’s ASHA Program

India’s accredited social health activists (ASHAs) are female CHWs selected from the village to which they belong (one per 1,000 population) to perform the following services: (1) counseling pregnant women and facilitating access to antenatal care (ANC) and facility delivery; (2) distributing oral rehydration packets, iron folic acid tablets, chloroquine, oral contraceptive pills, and condoms; (3) facilitating access to immunizations for children; and (4) providing information on health and health practices. The ASHA is required to have completed 8 years of education (9) but in places with severe human resource shortages this requirement has been relaxed, and women with no education but with demonstrable leadership skills have been selected. The ASHA works under the direct supervision of the ASHA facilitator and auxiliary nurse midwife (ANM) based at the closest sub-center. The program, which started in 2005, envisioned the ASHA as the first port of call for any health-related needs of marginalized sections of the population, especially women and children, who find it difficult to access health services.

Accredited social health activists receive incentives for the activities they carry out which range from Rs 50 (US$0.83) for early registration of pregnancy to Rs 1000 (US$16.67) for facilitating permanent contraceptive methods. ASHAs are also paid for identifying and referring cases of leprosy, tuberculosis, and malaria, undertaking health surveys, and mobilizing village health meetings. Incentives are disbursed from the ASHA program of the National Health Mission (NHM) of the Government of India and are augmented by other funds and schemes. One such scheme is the Jannai Suraksha Yojana (JSY) under which pregnant women are paid for delivering at an institution, and ASHAs are paid for mobilizing the women for the same. A working paper prepared for the NHM Advisory Council in 2011 recommended providing higher incentives for ASHAs as the current amount of payment was not proportional to the work required (10). Revision of incentive rates on current and new activities has been proposed by NHM so that ASHAs receive at least Rs 1000 ($16.67) a month (11). This has been implemented for certain routine procedures, such as attending monthly meetings and maintaining records.

ASHAs have expressed dissatisfaction with the incentives (10, 12) and expectations of better or regular pay (13). However, one study found no association between the level of dissatisfaction on incentives and motivation among ASHAs, indicating that incentives do not always drive motivation and work performance among this cadre of workers (14).

There are state variations in the incentive amounts paid to ASHAs. Certain states, such as Kerala, Haryana, and West Bengal, have decided to provide monthly fixed remuneration to ASHAs from their own budgets (15). However, it is unclear whether this has been implemented. In Haryana, the state government has implemented a fixed monthly remuneration of Rs 500 translating to U$8.30 (16), but anecdotal reports indicate that ASHAs have not been receiving this compensation. In Punjab, there is yet no fixed remuneration.

The broad objective of this qualitative study was to look at the influences of the family, community, and health system on the ASHA workers’ performance and motivation. Within each of these institutions, incentives play a role in directing the work activities of ASHAs. The current paper, therefore, deals with the role of incentives in motivating ASHAs and influencing their work. In addition, the paper seeks to document how ASHAs interpret information about incentives.

## Methods

Two districts, Gurdaspur and Mewat, were selected purposively because of their socio-economic and religious diversity. Gurdaspur, a district in Punjab, has lower than national average maternal mortality ratio (MMR) of 155 per 100,000 live births and infant mortality rate (IMR) of 26 per 1000 live births (17) compared to the national average of 178 per 100,000 and 40 per 1000 live births, respectively (18). However, Gurdaspur has a low child sex ratio (821 girls per 1000 boys) due to a cultural preference for male children. In rural areas of Gurdaspur, 79.5% of women deliver in facilities, 86.2% of women access any ANC, and full immunization coverage is 64.3% (17). Mewat, a district in Haryana, has a MMR of 160 per 100,000 live births and IMR of 64 per 1000 live births. In rural Mewat, 40.3% of women deliver in facilities, 30.5% women access any ANC, and full immunization has covered only 20.8% of children (19). According to the 2011 Indian Census, Mewat has a lower literacy rate (53 as compared to 80% in Gurdaspur), and lower work participation rate (27 compared to 33% in Gurdaspur) (18). Gurdaspur has 1253 ASHAs; Mewat has 908 ASHAs.

One block in each district was selected based on average performance in selected RMNCH + A indicators. In the selected block in Gurdaspur, 10 sub-centers were selected; five of which were high performing and five of which were poor performing in terms of RMNCH + A indicators. Two lists of ASHAs working in these two groups of sub-centers were compiled. The first 12 ASHAs from each list were selected. In Mewat, the selected block had four primary health centers (PHCs), each of which had between three and four sub-centers. One sub-center from each PHC was purposively selected to represent diverse caste and religious groups. Lists of ASHAs working in those sub-centers were compiled and the first six ASHAs on each list were selected for participation. One ASHA in the sample had no decision-making family member (e.g., husband or mother-in-law) living with her; therefore, we interviewed her son. However, we selected an additional ASHA who did have a decision-making relative living with her. A total of 49 eligible ASHAs were selected for participation in an interview, which provided a sufficiently large enough sample to achieve saturation. Inclusion criteria were: being married, below age 50 (while the official age limit of ASHA is 45 years, we assumed that there may be older women recruited as ASHA and, therefore, we wanted to keep the cut off age as 50), and consent to participate. Our final sample included 21 Sikhs, one Hindu, and three Christians in Gurdaspur, and nine Muslims and 15 Hindu in Mewat. Compared to ASHAs in Mewat, ASHAs in Gurdaspur spent a higher average number of years in school (9.7 compared to 6.5), had fewer children (two versus four), and had more experience as an ASHA (5 versus 2 years).

We also interviewed one family member for each interviewed ASHA: 34 husbands, 13 mothers-in-law, 1 sister-in-law, and 1 son. There was a preference to interview the husbands; however, in the event they were not available, the mother-in-law was interviewed. In two instances, neither the husband nor the mother-in-law was available and an alternative family member was interviewed (sister-in-law and son).

Trained interviewers conducted semi-structured interviews using a pre-tested guide. The first author conducted a 2-day training for interviewers on the purpose of the study, the interview guide, qualitative interviewing techniques, and human subjects research ethics. The interview guide was developed in English and then translated into Hindi and Punjabi and back translated into English. Following the interviewer training, the guide was pre-tested to ensure clarity of purpose and wording of the questions. The interviews were conducted in Hindi and Punjabi and audio-recorded with the permission of the respondents. Interviews were transcribed and translated into English. A sample of 15 transcripts was reviewed by two researchers and formed the basis of the coding scheme that was applied to the remaining transcripts. Codes related to incentives were then separated and examined individually as well as part of the entire narrative. Two key themes were identified and are discussed below: incentives as a source of power and independence and incentives as a cause of distress.

Written informed consent was obtained from all respondents. The study was approved by the Institutional Review Board of University Research Company (URC) in Maryland, USA and CMS Institutional Review Board, in Delhi, India.

## Findings

### Incentives as a Source of Power and Independence

Incentives lent some amount of self-reliance among ASHAs who reported an increase in self-confidence: “I always wanted that my work should get increased and I should earn more money. Then by earning more, my self-confidence gets increased. I feel encouraged to do more and more work.” (ASHA #5, Mewat)

This increased self-confidence was entwined with their growing financial independence. Earning their own money eliminated the requirement to rely on husbands and mothers-in-law for spending money.

I no longer have to please my relatives. Earlier I used to please them to get favours. Now I am working and earning my livelihood so I am no more under them. After becoming ASHA I don’t have to ask anybody for favours except God. I have gained strength. (ASHA #6, Mewat)

For most ASHAs, the finances gained from their work were spent on items for their children, with less being spent on themselves or their own family; most were able to use their own discretion on these types of purchases. Discussions around spending money on the ASHAs’ families, however, were still held with husbands or mothers-in-law with a joint decision-making process. An added benefit, reported by some ASHAs, was that there was less domestic conflict after they became ASHAs and started earning an income. There was also more sharing of household chores by mothers-in-law, husbands, and other female relatives, leaving time for ASHAs to accomplish their official duties. Additionally, in terms of decision-making in other areas of household management, such as health care seeking, ASHAs reported a shift as a result of their position. One ASHA explained:

Interviewer (I): Who takes decision for check-up if somebody falls sick at home?

Respondent (R): Yes, I give them advice to consult doctor for particular disease.

I: Did you give such advice before?

R: No, I started to give such advices after being ASHA worker. (ASHA #30, Gurdaspur)

Thus, working as an ASHA and gaining some financial independence and self-confidence were empowering for our respondents as it translated into household sharing of chores and increased decision-making around seeking health care. For some women, it led to decreased conflict with husbands.

### Incentives as a Cause of Distress

In spite of this developing self-reliance and independence, incentives were a cause of distress among ASHAs for several reasons.

#### Relative to Work Required

Accredited social health activists did not feel that incentives were commensurate with the responsibilities and level of effort required to complete the corresponding tasks. The majority of ASHA respondents in Gurdaspur complained that the amount of money they received was too low compared to their workload. This was not as common a complaint in Mewat, where being able to earn any income was important for the ASHA and her family. ASHAs were also required to complete forms documenting the services they provided, which functioned as invoices. The documentation was time consuming and presented a particular challenge for illiterate ASHAs, who sought help from their children, neighbors, or ANMs. Illiterate women sometimes were not paid because they did not fill in the forms correctly: “I am not being paid because I cannot fill the card correctly. Sometimes I do mistakes so I try to get it corrected by ANM by visiting the sub-center several times in a day” (ASHA #6, Mewat).

Accredited social health activists reported that some activities were bundled, requiring a level of effort not reflected in the corresponding compensation. For example, ASHAs were paid for attending institutional deliveries in government facilities. This could require a number of steps, including educating pregnant women about the benefit of delivering at facilities, arranging and accompanying them in the ambulance, and attending the entire delivery. Some ASHAs unable to attend deliveries at night reported that they failed to get paid for cases where they called an ambulance to transport a pregnant woman for delivery, but did not accompany her. This put pressure on them to attend night deliveries even if it meant leaving their own children, creating problems at home. In cases where pregnant women from the catchment area of ASHA returned to their natal home for childbirth, the ASHA was not paid for any activities related to ANC because she did not take the woman for delivery. Similarly, if a pregnant woman returned to her natal home in the ASHA’s village, the ASHA was paid only partially for linking the woman to institutional delivery. In one case, despite the time and effort expended on a pregnant woman, the latter used a private health facility at the time of delivery, depriving the ASHA of her incentive: “I want some facility like fixed salary. Because sometimes in a month, there is only one case, and sometimes at the time of delivery people go to private hospitals. So I didn’t get any incentive for my work” (ASHA #31, Gurdaspur).

#### Not Inclusive of All Job Aids

Incentives either did not cover or only partially covered tools ASHAs needed to effectively do their jobs. Incentives provided for specific items, such as purchasing a uniform, were not sufficient, with one ASHA commenting that it was “not enough to even buy a dupatta” (stole worn over the shirt or kurta) (ASHA #35, Gurdaspur). Payment for transport costs were provided only for some activities, such as taking pregnant women to facilities for delivery. However, ASHAs were also responsible for facilitating ANC, which included accompanying pregnant women and often involved traveling long distances. As one ASHA described,

You see I accompany a pregnant woman for 9 months and sometimes pay their journey fares also. Our village is 5 kms from health facility. There is no bus service. I have to take a rickshaw to visit health facility. The rickshaw puller takes Rs 20 [US$0.30] for one side and Rs 40 [US$0.70] for up and down. I spend on patient’s fare along with mine. (ASHA #33, Gurdaspur)

Accredited social health activists also reported using their own mobile phones for their work. They used their phones to call women to escort them for ANC and contacted them more frequently during the time nearing delivery to ascertain the status of the pregnant woman and call the ambulance. However, as this ASHA reports, their mobile phone charges were not covered, “We don’t get money for mobile phone, as we have to call patients and ANM frequently” (ASHA #49, Gurdaspur).

### Incentives as a Cause of Friction in Family and Community

Incentives were also paid inconsistently and, at times, were not paid in full, creating tension for some ASHAs with their families and communities. ASHAs faced criticism from their husbands, some of whom, especially those in Gurdaspur, wanted the ASHAs to leave the job. Some ASHAs claimed that they were allowed by their husband to continue work because the husbands expected the government to provide a regular salary in the near future. One husband reported,

Sometimes I feel bad as she does much work but don’t get much money. There is no fixed salary for her. Her incentives are less, sometimes I feel she should leave this job but I don’t ask her to do so because there is hope that their job may become regular and they will get fixed salary. (Husband #47, Gurdaspur)

The volume of documentation required both for the position and to receive incentives drew disapproval from husbands:

When I sometimes do paper work at night, then he [husband] says that the work is too much for an ASHA. He talks about the work load because now we are given very long forms for home visits also. It takes a lot of time. (ASHA #17, Mewat)

Neighbors also mocked the ASHAs for working for such low pay, as this ASHA described, “Sometimes other persons ask me about my salary. They don’t believe me when I say that I don’t get salary. Then they tell me why I am doing this work without salary. I feel bad about this.” (ASHA #48, Gurdaspur). This was echoed by another husband who commented, “They [people in the community] say that she doesn’t get a salary, she doesn’t get much money. Then why she is doing work for such a small amount of money?” (Husband #43, Gurdaspur).

Accredited social health activists reported that jealousy among community members often caused distrust of their motives, especially as payments incentivized particular services, such as taking a pregnant woman for institutional delivery and getting children immunized. In such cases, families could refuse to heed the advice of the ASHA, as this ASHA reported: “They [women in community] say, ‘what is the use of vaccination?’ You want your benefit, that is why you are coming to us.” (ASHA #4, Mewat). Jealousy of the neighbors affected ASHAs’ work, as one respondent reported, “Some of our neighbors go to houses of pregnant women and tell them not to go with me to hospitals…They feel jealous of our family. They can’t see progress of our family. So they don’t want my work to go well” (ASHA#33, Gurdaspur).

### Using Incentives to Drive Service Delivery

Certain activities may have been prioritized over others due to higher incentives. For example, incentives provided for permanent contraception methods were higher than other methods. In our study, ASHAs reported increased use of sterilization among women in Mewat and men in Gurdaspur. In Gurdaspur, husbands of ASHAs motivated other men and accompanied them to the facilities to receive vasectomies.

The way ASHAs interpreted messages about incentives may also have led them to focus on certain activities. While communicating about incentives, ANMs may have emphasized certain paid or higher paid activities to the detriment of others. ASHAs were not aware of many incentives other than those for institutional deliveries and immunization. The presentation of the incentives was misleading for women when deciding to apply for an ASHA position. For instance, when asked about whether she was told about compensation when she began her position, an ASHA from Gurdaspur said:

No. But she [ANM] told me that if baby is delivered by c-section [cesarean] then ASHA will get Rs 100 per delivery…Madam [ANM] also told me that salary would depend upon the delivery cases given to government hospitals. (ASHA #36, Gurdaspur)

This perceived prioritization of facility-based cesarean sections had been felt by the community. ASHAs expressed that the community believed that deliveries in institutions were mostly done through cesarean sections with little other option: “They blame us that doctors assist baby to be delivered by c-section intentionally, that they don’t wait for normal delivery” (ASHA #35, Gurdaspur).

## Discussion

Our findings revealed two distinct but connected patterns: (1) incentives provided a source of independent earning that was a cause of enhanced self-worth among ASHAs that motivated them to work further and (2) incentives were a cause of dissatisfaction and stress due to the nature of their amount, structure, disbursement, and pressure they placed on ASHAs’ relationships with family and community. Our findings are similar to other studies in which incentives for CHWs are often described as inadequate, irregular, delayed, and partly paid (3, 10, 12), affecting their motivation and performance (13, 20, 21). Incentives have been identified as a major limitation to ASHAs’ role as social change agents, as they tended to only engage in remunerated work while doing little to introduce social changes (20). In our study, we saw that ASHAs may have been pushing those services that were incentivized, such as female and male sterilization, which may have involved an element of coercion as has been found previously, where incentivizing one form of contraception led to its promotion over others, thus curtailing free and informed choice. However, since our study included only ASHAs and their family members, we do not have the community voice and cannot conclude anything definite.

Payment for CHWs has been a debated topic in the literature (3, 22–24). While incentives are a source of motivation (3, 25), there is little available data, as far as we are aware, on the link between financial incentives and sense of empowerment of CHWs in the public health literature. We found only one study conducted among health program managers that mentions the potential of economic empowerment as a source of work effectiveness among CHWs (22). Our findings document ASHAs’ expressions of empowerment resulting from the financial independence afforded by incentives and the individual transformations that accompany poor women entering wage employment (26, 27). The ASHA program, in this context, is said to be providing a double benefit – that of making health care accessible to the community as well as providing jobs and remuneration to the female section of the community, thus widening the scope for decision-making and gender equality among previously disempowered women.

Accredited social health activists in our study reported dissatisfaction with part payments and often had no knowledge of how much would be paid for what work. Therefore, it is not surprising that they wanted to be paid a consolidated amount rather than in part payment (28). They also expressed dissatisfaction that the incentives paid were not commensurate with the work expected, echoing other findings that these did not reflect their abilities or level of responsibility (29, 30).

Our study had an added dimension in that we also interviewed family members of ASHAs who reported similar dissatisfaction with disproportionate incentives and felt that the ASHAs in their families worked excessively. ASHAs depended on the goodwill of their husbands and families to continue work; disapproval might cause them to be less effective in their work. Many ASHAs were able to maintain their positions with family support because of their spouses’ belief and hope that, in future, ASHAs would receive a regular salary. Without movement in this direction, it is possible that there will be attrition among ASHAs, particularly in a wealthier district such as Gurdaspur. Our findings on community mistrust of ASHAs’ motives are also in line with other studies (3, 7), further warranting support and public recognition of ASHAs by health and local government authorities to dispel such doubts among community members.

The way ASHAs interpreted messages about incentives is a unique finding. We saw that communications about which activities would be paid informed the understandings of job responsibilities. This is an important area of research as our current understanding of communication between health system supervisors and CHWs is limited. Women known to the ANM or others in the health system structure are selected for the position (12, 31). To encourage women to take the role, supervisors may provide partial or biased information about paid activities. This emphasis could then be interpreted by the ASHA as the best activity to be practiced or promoted, such as cesarean sections.

This study did not explore whether ASHAs targeted those who were easier to reach in an effort to obtain incentives with less effort. In general, it is found that women with higher household income, education, and lower parity utilize maternal and child health services more than those who are poor, have low education, and higher parity (28, 32–35). It is possible that ASHAs, in their hurry to achieve targets for which they are remunerated, may not focus on sections of the community that are more difficult to persuade. This could be an area for further study.

We have to interpret these findings with caution as there are limitations in our study. The focus of this study was not solely on incentives. The findings presented here are based on the ASHAs’ reports on topics tangential to incentives and income, such as challenges they experienced in the performance of their duties. Interviews may not have explored the topic of incentives as thoroughly as if that had been the focus of the study. However, our findings do point the way for future research that can contribute to more nuanced understandings of incentives in CHW programs.

## Conclusion

Incentives, while contributing to motivation, financial independence, and self-confidence among ASHAs, come with many caveats around appropriate, acceptable, and timely remuneration for activities and the ASHA relationship with her family and community, including her supervisors. Future areas of research include examining the effect of incentives on whether communities are receiving unbiased information about health activities, whether ASHAs preferentially target higher-income members of the community, and how community members feel about the information and advice they receive from the ASHA. Given the limitations in our study, we tentatively suggest the following recommendations: all efforts need to be made to ensure timely payments of incentives so that a clear link is established between work done and incentive received. Furthermore, efforts should be made to “unbundle” incentives associated with different aspects of care to ensure that ASHAs are compensated for and perform every step in the process. Incentive schemes should also accurately reflect the out-of-pocket costs associated with the position, such as transportation and mobile phone costs. There have been certain revisions in the incentive structure in both Punjab and Haryana since our study. One of this is Closed User Group (CUG) connections provided to ASHAs to communicate with fellow ASHAs, ANMs, or PHCs free of cost and some credits (INR 50 in Punjab, INR 100 in Haryana) for calling outside the CUG (36). Similarly, information about incentives should be communicated clearly to all those involved in program implementation and most importantly, to ASHAs themselves. This information should be presented in such a way as to not favor activities with higher incentives over other activities. The emphasis should be placed on providing quality and appropriate services to all clients. Additionally, documentation associated with securing incentives should be revised to reduce unnecessary burden on ASHAs, including adjusting the documents to be inclusive of all literacy levels. Finally, although our findings and recommendations are specific to the Indian context, they can be informative to incentivized programs of CHWs in other low and middle income country settings as they face similar issues.

## Author Contributions

ES was PI of the study, supervised data collection, conducted analysis, developed the structure of paper, conducted literature review, wrote major sections, and wrote up findings. SSL was co-PI of the study, reviewed data, collaborated in analysis, contributed to writing discussions and findings, and structured bibliography. AS helped in writing recommendations, reviewed findings in light of field reality, provided information on incentive schemes for state of Punjab, and provided references for same. SR reviewed findings in light of field reality, provided information on incentive schemes for state of Haryana, provided references for same, examined and verified findings specific to Haryana state, and contributed to writing of recommendations NL provided guidance on design and implementation and contributed to the discussion, conclusions and recommendations.

## Conflict of Interest Statement

The authors declare that the research was conducted in the absence of any commercial or financial relationships that could be construed as a potential conflict of interest.
